# Practices in the prescription of antiseizure medications: is it time to change?

**DOI:** 10.1055/s-0043-1777806

**Published:** 2024-03-26

**Authors:** Lécio Figueira Pinto, Lucas Scárdua Silva, Rafael Batista João, Vinícius Boldrini, Fernando Cendes, Clarissa Lin Yasuda

**Affiliations:** 1 Universidade de São Paulo, Faculdade de Medicina, Hospital das Clínicas, Instituto Central, Divisão de Clínica Neurológica, São Paulo SP, Brazil.; 2 Universidade de São Paulo, Faculdade de Medicina, Hospital das Clínicas, Instituto de Psiquiatria, Programa de Neuropsiquiatria (PROJEPSI) São Paulo SP, Brazil.; 3 Universidade Estadual de Campinas, Brazilian Institute of Neuroscience and Neurotechnology (BRAINN), Campinas SP, Brazil.; 4 Universidade Estadual de Campinas, Faculdade de Ciências Médicas, Departamento de Neurologia, Campinas SP, Brazil.

**Keywords:** Epilepsy, Therapeutics, Anticonvulsants, Epilepsia, Terapêutica, Anticonvulsivantes

## Abstract

The treatment of epilepsy has advanced over the past 30 years through the development of new antiseizure medications (ASMs). Unfortunately, not all of them have been approved yet in Brazil, and many are still underused. When comparing new ASMs to older ones, they are generally not more effective in treating epilepsy. However, they offer better tolerability, with fewer interactions and long-term side effects, especially for patients with comorbidities or those requiring polytherapy. Enzyme induction caused by older ASMs is associated with increased cholesterol levels, drug interactions with decreased effects of statins and other cardiovascular medications, anticoagulants, chemotherapy, immunosuppressors, anti-infective agents (including HIV treatment), antidepressants, and contraceptives. Additionally, they can reduce levels of vitamin D and sex hormones, as well as decrease bone density. The increasing concern about these effects during life, especially after prolonged exposure, has led most developed countries to change prescription patterns in favor of new ASMs, particularly levetiracetam and lamotrigine. Both are also considered the safest options for women of childbearing age. Regrettably, the prescription trends in Brazil have remained largely unchanged over time. This can be partially attributed to the slower approval process of ASM and the reluctance of general physicians and neurologists to embrace these new concepts. In this concise review, we highlight the various advantages linked to the new ASM, aiming to promote a shift in the prescription pattern for ASM. The selection of ASM should be customized according to individual characteristics, and practical suggestions for choosing ASMs are provided in this paper.

## Resumo

O tratamento da epilepsia avançou nos últimos 30 anos com o desenvolvimento de novos medicamentos anticrise (MAC). Infelizmente, nem todos estão aprovados no Brasil e muitos ainda são subutilizados. Os novos MAC não são mais eficazes que os antigos, mas apresentam melhor tolerabilidade, menos interações e efeitos colaterais a longo prazo, especialmente para pacientes com comorbidades ou que necessitam de politerapia. A indução enzimática causada pelos MAC antigos está associada ao aumento dos níveis de colesterol, interações medicamentosas com redução do efeito das estatinas e outros medicamentos cardiovasculares, anticoagulantes, quimioterapia, imunossupressores, agentes anti-infecciosos (incluindo tratamento do HIV), antidepressivos e contraceptivos. Além disso, podem reduzir os níveis de vitamina D e hormônios sexuais, podendo afetar a massa óssea. A crescente preocupação sobre estes efeitos ao longo da vida, com a exposição prolongada, levou a maioria dos países desenvolvidos a modificar o padrão de prescrição com maior uso dos novos MAC, especialmente levetiracetam e lamotrigina. Ambos são considerados as opções mais seguras para mulheres em idade fértil. Infelizmente, as tendências de prescrição no Brasil permaneceram praticamente inalteradas ao longo do tempo. Isto pode ser parcialmente explicado pela lentidão no processo de aprovação dos MAC e à resistência dos médicos generalistas e neurologistas em adotar estes novos conceitos. Nesta revisão, destacamos as vantagens dos novos MAC e a necessidade da mudança no padrão de prescrição também no Brasil. A escolha do MAC deve ser feita de acordo com as características individuais dos pacientes e sugestões práticas são apresentadas.

## INTRODUCTION

The comprehensive treatment of people with epilepsy (PWE) should strive to offer a life free from the constraints associated with epilepsy. As recently mentioned by the World Health Organization (WHO) in establishing the Intersectoral Global Plan on Epilepsy and other neurological diseases (IGAP), managing seizures is just one aspect of treating this long-term illness. One of the strategic objectives of this global plan is to ensure that individuals have access to appropriate anti-seizure medications (ASM) based on their specific requirements (such as children, adolescents, and women of childbearing age). Unfortunately, PWE living in low- and middle-income countries face greater concerns due to difficult access to services for epilepsy and anti-seizure medications (ASM). These factors, combined with misconceptions, stigma, and lack of knowledge, result in treatment gaps and a disproportionate burden for patients, families, and society. Part of the treatment gap results from a lack of information and misconceptions related to ASMs.

The development of new antiseizure medications (ASMs) in the last thirty years created new possibilities in treating epilepsy, considering the different profiles of pharmacodynamics and pharmacokinetics. While only some of the newer ASMs have been approved in Brazil, they are not being utilized to their full potential. Different studies have broadly discussed the positive impact of these new ASMs, resulting in significant changes in treatment rationale and clinical practice worldwide. Most benefits are associated with improved quality of life, less drug interaction, reduced impact on cognition (and comorbidities), and fewer adverse effects. 1 2 3 4 5 6 7 8

Unfortunately, little discussion has been raised in Brazil about the worldwide changes in ASM usage. Expanding the knowledge about the new ASMs from a practical point of view may help physicians change their old perspectives to offer newer ASM alternatives for PWE. Table 1 shows the older and newer ASMs considered in this discussion.

**Table 1:** Older and new antiseizure medications

Older	Newer
Phenobarbital Phenytoin Primidone Carbamazepine Valproate	Oxcarbazepine Lamotrigine Topiramate Gabapentin and Pregabalin Vigabatrin Levetiracetam Lacosamide Perampanel Cannabidiol

Note: *Although cannabidiol is not a new medication, its use for some epilepsy syndromes (i.e., Lennox-Gastaut and Dravet Syndrome) has been established more recently.

## PHARMACOLOGICAL ASPECTS

### Are the new ASMs better than the older ones?

How can we compare the ASMs? One specific ASM can be considered superior due to a combination of different aspects, including higher efficacy, tolerability, safety, and retention rates. As efficacy is the main result in clinical trials, it should be the focus to start the evaluation of a specific ASM.

**Figure 1:**
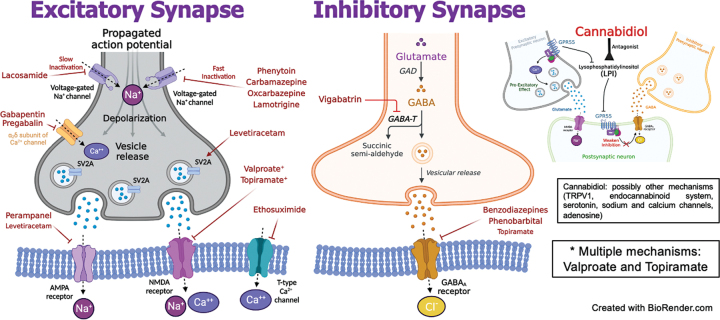
Mechanisms of action of antiseizure medications available in Brazil (Adapted from 48 49 50 ).

**Figure 2:**
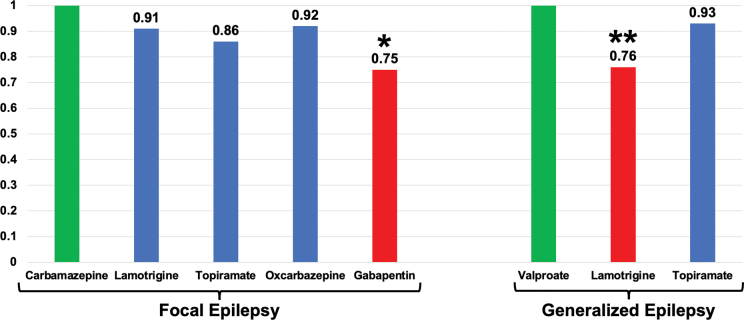
Note: *Lamotrigine, Topiramate and Oxcarbazepine did not differ from Carbamazepine, while *Gabapentin was less effective than carbamazepine for focal epilepsies. ** Lamotrigine was less effective than valproate for generalized epilepsies, while there was no significant difference between valproate and topiramate. Adapted from. 15 16 Comparison of efficacy of antiseizure medications for focal (the newer ASMs were compared to carbamazepine) and generalized epilepsy (the newer ASMs were compared to valproate).

Previous studies comparing newer with older ASMs showed no differences in terms of efficacy. 5 9 Despite the development of new ASMs and different mechanisms of action ( Figure 1 ), seizure control is similar to the older ASMs. 7 10 There are few studies evaluating the head-to-head efficacy of ASMs. Recent evidence comes from the SANAD (Standard and New Antiepileptic Drugs) studies conducted in the United Kingdom for focal and generalized epilepsies. Data from these studies are presented in Figure 2 . There is no evidence of improved efficacy of the newer ASMs for focal epilepsy. 11 12 13

In terms of efficacy, it is essential to consider seizure type. It has been recognized that some ASMs are not appropriate for generalized seizures. The most striking example is juvenile myoclonic epilepsy (JME), as myoclonic and absence seizures may worsen with carbamazepine, oxcarbazepine, and phenytoin. Valproate has proven to be the best choice for generalized epilepsies, surpassing lamotrigine, levetiracetam, and topiramate. 13 However, extreme caution is necessary when treating women of childbearing age due to increased risks of teratogenesis associated with valproate. 14

### So, what are the advantages of the new ASM?

One of the main advantages of newer ASMs is their improved tolerability and safety profiles. While the older ASMs (such as phenobarbital, phenytoin, and carbamazepine) are associated with a significant risk of long-term clinical side effects, the newer ASMs are generally better tolerated and have fewer interactions with other drugs. This improved profile can be particularly beneficial for patients with comorbidities or those requiring polytherapy. 15 16

### Enzyme induction effects

**Figure 3:**
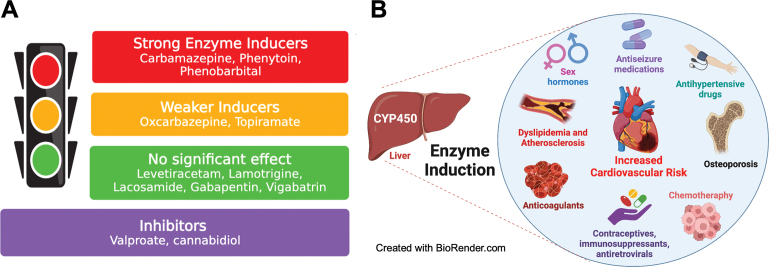
Relationship between antiseizure medications and Enzyme induction ( **A** ) (Adapted from 24 51 ). ( **B** ) Negative effects of enzyme induction due to drug interactions and metabolic effects (Adapted from 8 18 24 35 36 ).

Studies suggest that enzyme induction should be a concern in epilepsy treatment. 17 Older ASMs are more frequently associated with the enzyme induction phenomenon (usually linked to the cytochrome P450 enzyme induction) than the newer ones ( Figure 3A ). Enzyme induction is an essential factor to be considered in ASM selection for PWE due to the problems associated with drug interaction and metabolic effects. 8 12 13

Enzyme induction is associated with increased cholesterol levels and decreased effects of statins and other cardiovascular medications. These changes, added to factors such as reduced physical activity and other unhealthy lifestyle habits, may increase the cardiovascular risk of PWE. 18 19 20 One recent study showed a 21% increase in risk for individuals who used enzyme inducers ASMs after ten years of exposure. 21 Problems related to enzyme induction are not limited to cardiovascular effects. There is a reduction of vitamin D and bone mass density, which results in the early occurrence of osteopenia and osteoporosis and an increased risk of fractures. Besides, the enzyme inducers may decrease the sex hormones, which negatively impact the bone mass and cause sexual dysfunction. 22 Another investigation demonstrated normalization of the levels of testosterone, progesterone, cholesterol, and low-density lipoprotein after switching from carbamazepine to lacosamide as adjunctive therapy to levetiracetam (based on a cross titration over four weeks, followed by an 8-week maintenance period). 23

There is also a reduction in the effect of other medications, including anticoagulants, chemotherapy, immunosuppressors, anti-infective agents (including HIV treatment), and contraceptives. Reduced levels of various medications can cause serious issues, ranging from undesired pregnancies to ineffective chemotherapy and the progression of cancer. Figure 3B shows some of these negative aspects of the enzyme-inducing ASMs. 4 24 25

### Teratogenesis

Another major issue with the ASMs is related to the treatment of women of reproductive age. Some ASMs should be avoided due to the increased risks of teratogenicity, cognitive impairment, learning deficits, increased risk of autism spectrum disorder, and attention deficit hyperactivity disorder in children exposed to some ASM intrauterus. 26 27

**Figure 4:**
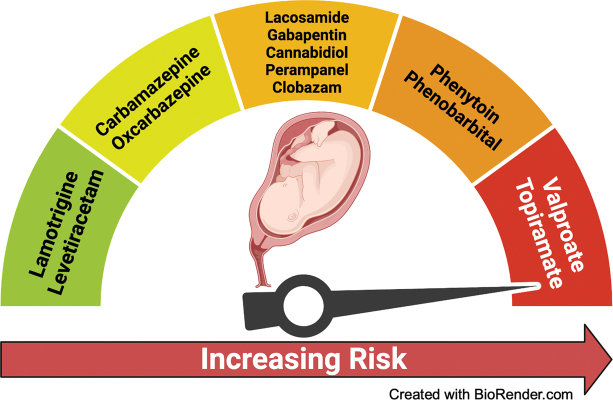
Illustration of the teratogenic risk profile of antiseizure medications (Adapted from 26 27 ).

Some newer agents, particularly levetiracetam and lamotrigine, are considered safe for women of childbearing age. Some older ASMs, such as oxcarbazepine and carbamazepine, also showed reassuring safety data. 27 Conversely, valproate, and topiramate have been repeatedly associated with an increased risk of teratogenicity, followed by phenobarbital and phenytoin. Unfortunately, there is insufficient data related to teratogenicity for many of the newer ASMs, including lacosamide, perampanel, clobazam, and cannabidiol ( Figure 4 ). 26 27

### Tolerability

Although the efficacy of new and old ASMs are similar when the medication is adequate for the seizure type, the tolerability may vary according to individual characteristics and comorbidities. Personal lifestyle and comorbidities need to be accounted for when choosing an ASM. For example, levetiracetam should be avoided for individuals with a history of anxiety, depression, and other psychiatric disorders; likewise, valproate should be avoided for patients with obesity. On the contrary, some individuals may benefit from topiramate's effect for weight loss (as long as they do not present glaucoma or nephrolithiasis). Some ASMs present a neutral profile regarding the impact on cognition (such as levetiracetam and lamotrigine), while others may cause significant cognitive dysfunction (phenobarbital and topiramate). Some of these potential effects of ASMs are presented in Table 2 .

**Table 2:** Characteristics of Potential adverse effects and comorbidities associated with ASMs (Adapted). 1 2 6 28 44 45 46 47

Abbreviations: PB, phenobarbital; PHT, phenytoin, TPM, topiramate; BZD, benzodiazepines, such as clobazam, clonazepam; LTG, lamotrigine; LEV, levetiracetam; LCM, lacosamide; CBZ, carbamazepine; OXC, oxcarbazepine; PER, perampanel; GBP, gabapentin; PGB, pregabalin.

As illustrated in Table 2 , there is no ideal ASM (i.e., without the potential to cause adverse effects). Although there are various profiles of mechanisms of action ( Figure 1 ) and side effects, in general, there are no major differences in tolerability among the new ASM. 28 Out of the newer ASMs, topiramate and oxcarbazepine have the highest likelihood of causing intolerable side effects, which result in the earlier discontinuation of these medications. 28

## PRESCRIPTION PATTERNS AND CHALLENGES

### What is happening in Brazil?

**Figure 5:**
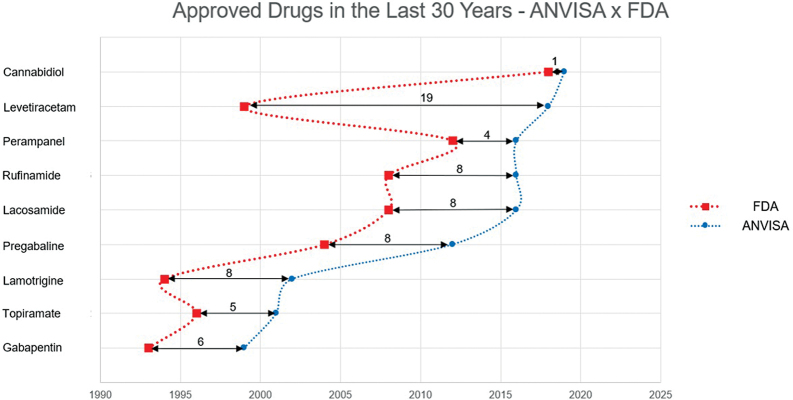
The chronological evolution of ASMs approval by the Food and Drug Administration (FDA) (red squares) and the Brazilian Health Regulatory Agency (ANVISA, blue dots) in the last 30 years. Only two ASMs (oxcarbazepine and vigabatrin) were approved by ANVISA before the FDA in this period. 52 53

Unfortunately, the ASḾs approval in Brazil usually happens several years after the initial consent in the USA (FDA) and Europe (EMA) ( Figure 5 ). One striking example is Levetiracetam, whose approval was delayed 19 years, preventing several patients from benefiting from this ASM. While both the US and Europe already have access to other newer ASMs, we remained mainly restricted to relatively older drugs in Brazil. These delays have hindered access to newer ASMs for decades, leading to less desirable prescription patterns with the frequent use of enzyme-inducing ASMs. 29 As physicians tend to use medications they are more acquainted with, faster approval is desirable to generate an earlier comprehension of the characteristics of the newer drugs and, consequently, faster construction of more appropriate protocols. All these difficulties impair an adequate treatment for PWE.

Although many of the new-generation ASMs are currently available in Brazil (i.e., lamotrigine, levetiracetam, lacosamide, perampanel, pregabalin, gabapentin, vigabatrin, oxcarbazepine, topiramate, rufinamide, and cannabidiol), most are not distributed by the Brazilian unified health system (Sistema Único de Saúde [SUS]). The SUS provides (free of charge to the population) the first-generation ASMs clonazepam, valproate, as well as the enzyme-inducer ASMs [EI-ASMs] phenobarbital, phenytoin, and carbamazepine at the primary care health units. In addition, some of the newer ASMs (lamotrigine, levetiracetam, topiramate, vigabatrin, and gabapentin), and clobazam may also be obtained through SUS; however, requiring much paperwork and bureaucracy from physicians and associates, with several barriers for patients and caregivers to reach the specialized pharmacy dispensaries.

PWE often face challenges in obtaining the correct prescription (along with the necessary paperwork) and accessing specialized dispensaries. Additionally, they frequently experience frustration due to the inadequate availability of proper anti-seizure medication at these specialized centers. 30 Adding to the challenges of accessing newer ASMs, there are only a limited number of specialized epilepsy centers in the country, mostly in large cities. PWE from smaller towns are seldom prescribed and face several obstacles in obtaining newer medications.

### What is happening in the world?

**Figure 6:**
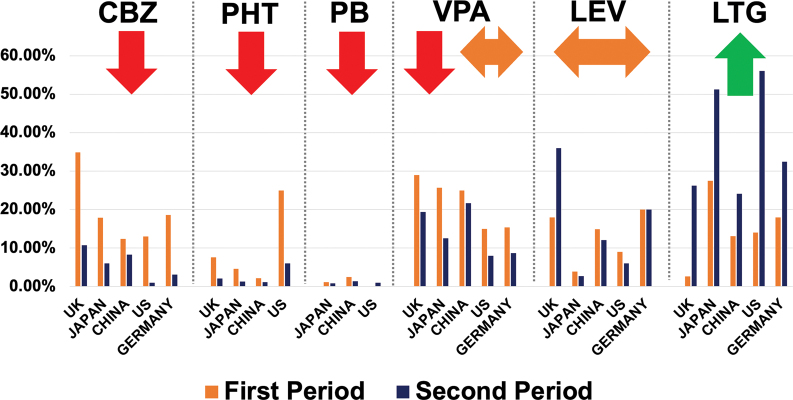
Changes in prescribing patterns over time for antiseizure medications in different countries followed by the time period evaluated: United Kingdom (UK: 2003-2016), Japan (2015-2018), China (2013-2018), United States (US: 2012-2019) and Germany (2008-2020). (Figure adapted from 31 54 55 56 57 58 :).

Different studies have demonstrated a clear trend toward using newer ASMs in many countries in the last years, as illustrated in Figure 6 . Some countries have reduced the use of carbamazepine and phenytoin in favor of increasing levetiracetam and lamotrigine. 31 These changes have been driven mainly by better safety and tolerability profiles, with less enzyme induction activity.

Interestingly, using changes of ASM prescription practices in the UK as an example did not lead to an increase in cost. The median standardized monthly direct health care cost was £229 for the EI-ASMs and £188 for the non-EI-ASM cohorts. The median cost was higher for the EI-ASMs cohort in every year of follow-up, and the median time to treatment failure was also shorter in the EI-ASM cohort (468 vs. 1194 days). Based on their findings, the authors suggested that changing treatment practices could potentially improve patient outcomes and reduce overall costs. 32 This is likely because there are more complications associated with the use of outdated drugs, requiring more frequent laboratory tests, as well as the need for vitamin and hormonal supplementation. Additionally, there is a requirement for increased dosages of concomitant medications, as their serum levels are reduced by EI-ASM.

Another study from Germany also showed a decrease in the prices of new ASMs, while the overall expenses remained stable, despite an increase in the prescription of newer and non-enzyme-inducing medications for PWE. 33

### Should Brazil start changing its prescription pattern?

Choosing an ASM for a PWE is the next step after a proper diagnosis. This choice is crucial because the chances of being seizure-free after the failure of the first two ASM regimens is only around 10%. 34

Most people who started on treatment will continue to use ASMs for many years, eventually, for the rest of their lives. Therefore, a personalized choice requires balancing efficacy, long-term effects, tolerability, and safety. These ideas align with the objective of offering an integral treatment for PWE: controlling seizures and avoiding adverse effects and long-term problems.

Considering all the available evidence, we believe there is a need for a change in the prescription pattern in Brazil. While the delay in approving the newer ASMs has hindered and postponed these changes for decades in Brazil (with the extreme example of levetiracetam, which could have benefited many patients with focal and generalized epilepsies), the current presence of newer ASMs in the Brazilian market has been insufficient to motivate neurologists and non-neurologists to change their pattern of prescriptions. Several factors may contribute to the persistent trend of prescribing outdated ASMs, particularly the enzyme inducers. Examples of such ASMs include the 58-year-old carbamazepine, the 85-year-old phenytoin, and the 111-year-old phenobarbital. Firstly, many physicians are familiar with and accustomed to these ancient drugs. Secondly, these medications are relatively inexpensive and more easily accessible at primary care facilities. There are other reasons why physicians may be hesitant to modify their prescriptions. These include misconceptions and a lack of understanding regarding the advantages of newer ASMs. Additionally, obtaining the newer ASMs can be quite challenging due to excessive paperwork and limited dispensary centers, as discussed below.

Moreover, the newer ASMs often come with higher price tags and may not be covered by the public healthcare system.

## ACCESS TO ASMs IN BRAZILIAN PUBLIC HEALTH SYSTEM: PAPERWORK, BUREAUCRACY, AND LACK OF AVAILABILITY

The vast majority of PWE in Brazil depend upon ASMs provided by the public system, and the oldest medications are usually the only ones available. Not only that, but the law dictates that the cheapest formulation should be purchased and distributed by the public system, regardless of the “quality” of the product. It is important to acknowledge that seizure control may be lost when generic and other brand formulations are provided by the public health system and there is a need to review the process and ensure that bioequivalence and other pharmaceutical aspects of the medications purchased by the government reach the desired standard.

Therefore, in many instances, the main problem is not that neurologists ignore the advantages of newer ASMs, but rather that they have no choice other than to prescribe the old drugs. The Clinical Protocol and Therapeutic Guidelines for Epilepsy (PCDT – Protocolo Clínico e Diretrizes Terapêuticas para Epilepsia) implemented by the Brazilian government has many issues. The old drugs are considered first-line medications, and the newer ones are available only for switch after failure.

Many factors need immediate attention. First, from a medical standpoint, it is necessary to provide nationwide ongoing education to spread scientific knowledge and motivate both young and experienced doctors to tailor their prescriptions based on individual patient characteristics and requirements. Secondly, there is an urgent need to streamline administrative processes and facilitate access to the latest ASMs within the public healthcare system. There is a need to diminish the paperwork needed to provide newer medications for PWE. There is no point in requiring new forms when patients have been obtaining ASMs month after month. The diagnosis is clear and requires continuous treatment for seizure control. The bureaucracy is a step with no obvious reason that just limits access for PWE.

Newer ASMs should be available as first-line therapy in the public health system for the reasons discussed previously. Furthermore, under current rules, the use of two new ASMs as polytherapy is not permitted. There is no scientific basis for this, and it ends up being another barrier to better treatments for PWE in the Brazilian public system.

The current system is not user-friendly for PWE, especially considering that some may have cognitive impairments, memory issues, and other additional medical conditions, while many are unemployed, unable to drive, and face difficulties with public transportation. Unfortunately, the centers are not evenly distributed throughout the country, and ASMs are often unavailable.

There is a lack of continuous availability of ASMs in the public health dispensary, and the official bureaucracy installed by the health system are reasons that contribute to the non-prescription of these new-generation ASMs. Therefore, our medical societies must educate and pressure government authorities on this topic, with the support of society, especially PWE, families, and patient associations.

We hope this short review raises the attention to the importance of different aspects related to the care of PWE. Among the different problems PWE encounter, some can be avoided with an appropriate choice of ASM and better access to treatments.

## PRACTICAL RECOMMENDATIONS FOR CHOOSING ASMs

In a country as big as this, there are areas where numerous patients face difficulty in receiving proper medical care and struggle due to a lack of diagnosis. On the other hand, there are other regions where individuals with accurately diagnosed epilepsies could have access to newer ASMs, but most physicians choose to stick with outdated prescription practices. Here, we provide a few suggestions for physicians who are not specialists but are responsible for treating patients with epilepsy in an outpatient setting:

- Personalize the ASM selection based on epilepsy type, age, gender, drug interaction, side effects, and comorbidities profile; rationale: the most suitable medication should be selected for each patient, as some patients may experience greater benefits or harm based on clinical profile. For instance, patients with arrhythmia may experience harm from sodium channel blockers. Those with psychiatric symptoms may deteriorate if given levetiracetam ( Table 1 ), and carbamazepine and phenytoin may increase seizure frequency in patients with primary generalized epilepsies such as JME. 12 13 Therefore, levetiracetam for PWE with arrhythmia, lamotrigine for PWE with epilepsy and psychiatric issues and valproate for men with JME would be better choices.
- Prefer non-EI-ASM for newly-diagnosed patients. Rationale: as previously described, non-EI-ASMs are related to better adherence, tolerability, and quality of life and fewer long-term side effects and drug interactions ( Figure 3 ). 8 18 24 35 36
- After choosing an appropriate ASM according to the seizure type, consider exploring it to the maximum tolerable doses instead of using many ASMs in low doses. Avoid the association of multiple ASMs. Rarely use three ASMs, almost never four, and never more than that; rationale: monotherapy is commonly a better choice as it reduces drug interactions and side effects and maximizes adherence. 37 38
- In PWE and comorbidities, consider choosing a unique ASM for treating epilepsy and the comorbidities (e.g., lamotrigine or valproate to treat psychiatric symptoms); rationale: as in item 3, the fewer medications, the better.
- Be aware of common potential drug interactions, especially for EI-ASM (phenytoin, carbamazepine, phenobarbital, topiramate, oxcarbazepine, and primidone) and inhibitors (valproate and cannabidiol); rationale: pharmacodynamic and pharmacokinetic drug interactions can potentially cause loss of efficacy and intoxication (both for ASM-ASM and ASM-other drugs interactions – as contraceptives, anticoagulants, and others) ( Figure 3 ). 8 18 35 36
- Avoid valproate, topiramate, phenobarbital, and phenytoin for women of childbearing age; rationale: they are highly teratogenic and should not be prescribed for childbearing-age women ( Figure 4 ). 26 27
- In the absence of newer ASMs, prefer carbamazepine (for focal epilepsies), valproate (for generalized or unknown onset), and benzodiazepines (as adjunctive ASM). Avoid prescribing phenobarbital, primidone, and phenytoin; rationale: phenobarbital, primidone, and phenytoin have a considerable number of chronic irreversible side effects, such as cerebellar atrophy, gingival hyperplasia, osteoporosis, and connective tissue disorders. 6 20 39
- Avoid combining ASMs with similar mechanisms of action (e.g., lamotrigine and carbamazepine, lacosamide, oxcarbazepine, and phenytoin); rationale: it usually does not yield better seizure control and may potentiate the side effects. The combination of ASMs with different mechanisms (such as lamotrigine + levetiracetam or clobazam) may improve the chances of seizure control and reduce adverse reactions. 40
- Especially in the presence of chronic comorbidities (osteoporosis, high cardiovascular risk, infections, transplants, autoimmune diseases, cancer), consider referencing EI-ASM users for an epilepsy specialist; rationale: as far as we know, no studies in the literature evaluate the impacts of changing EI-ASM to non-EI-ASM over time. However, patients at high risk of side effects or drug interactions may benefit from change. Considering the side effects and the seizure risks related to scheme modifications, it is reasonable to refer the patient to an epilepsy specialist.
- Frequently and actively access side effects (especially those not usually reported, like sexual dysfunction); rationale: PWE frequently suffer side effects from ASMs (especially older ASMs). 41 An adequate treatment of side effects (which may include changing the ASM) may improve the quality of life and adherence ( Table 2 ).

In conclusion, this review focused on PWE that will start a medication. Changing ongoing epilepsy treatments is often complex and risky and should be performed in specific situations after assessing the risk-benefit ratio. ASM choices for PWE starting treatment should be personalized, considering seizure type (different efficacy profile), age, gender, and comorbidities. Choices should not be based on immediate cost alone but on overall cost-benefit. Recent data show that newer ASMs are generally better choices due to the lack of enzyme induction, drug interaction, and safety in women of childbearing age. 10 11

Worldwide evidence and prescription patterns have changed and point to lamotrigine and levetiracetam as the best options for treating epilepsy. Lacosamide is an attractive option but presents a higher cost and is currently unavailable in the public health system. Valproate is the most effective ASM for generalized epilepsies 42 ; however, its teratogenic potential poses a risk for women of childbearing age, limiting, but not excluding, its use. 43 We hope that the prescription pattern in Brazil will change, reflecting better care for people with epilepsy, based on the availability of new ASMs in recent years and the possibility of obtaining them in public health system dispensaries.
